# The Effectiveness of Deep Learning in the Differential Diagnosis of Hemorrhagic Transformation and Contrast Accumulation After Endovascular Thrombectomy in Acute Ischemic Stroke Patients

**DOI:** 10.3390/diagnostics15091080

**Published:** 2025-04-24

**Authors:** Mehmet Beyazal, Merve Solak, Murat Tören, Berkutay Asan, Esat Kaba, Fatma Beyazal Çeliker

**Affiliations:** 1 Department of Radiology, Recep Tayyip Erdogan University, Rize 53100, Turkey; drbeyazal@hotmail.com (M.B.); esatkaba04@gmail.com (E.K.); fatma.bceliker@erdogan.edu.tr (F.B.Ç.); 2 Department of Electrical and Electronics Engineering, Recep Tayyip Erdogan University, Rize 53100, Turkey; murat.toren@erdogan.edu.tr (M.T.); berkutay_asan21@erdogan.edu.tr (B.A.)

**Keywords:** contrast accumulation, endovascular treatment, hemorrhagic transformation, stroke, deep learning, artificial intelligence, neuroimaging, stroke imaging

## Abstract

**Objectives**: Differentiation of hyperdense areas on non-contrast computed tomography (NCCT) images as hemorrhagic transformation (HT) and contrast accumulation (CA) after endovascular thrombectomy (EVT) in acute ischemic stroke (AIS) patients are critical for early antiplatelet and anticoagulant therapy. This study aimed to predict HT and CA on initial NCCT using deep learning. **Material and Methods**: This study was conducted between January and December 2024. The study included 556 images of 52 patients (21 female and 31 male) who underwent EVT due to AIS, with hyperdense areas observed in the NCCT examination within the first 24 h post-EVT. The evaluated images were labeled as ‘contrast accumulation’ and ‘hemorrhagic transformation’. These labeled images were trained with nine different models under a convolutional neural network (CNN) architecture using a large dataset, such as ImageNet. These models are DenseNet201, InceptionResNet, InceptionV3, NASNetLarge, ResNet50, ResNet101, VGG16, VGG19 and Xception. After training the CNN models, their performance was evaluated using accuracy, loss, validation accuracy, validation loss, F1 score, Receiver Operating Characteristic (ROC) Curve, confusion matrix, confidence interval, and *p*-value analysis. **Results**: The models trained in the study were derived from 556 images in data sets obtained from 52 patients; 186 images in training data for CA and 186 images training data for HT (with an increase to 558 images), 115 images used for validation data, and 69 images were compared using test data. In the test set, the Area Under the Curve (AUC) metrics showing sensitivity and specificity values under different cutoff points for the models were as follows: DenseNet201 model AUC = 0.95, InceptionV3 model AUC = 0.93, NasNetLarge model AUC = 0.89, Xception model AUC = 0.91, Inception_ResNet model AUC = 0.84, Resnet50 and Resnet101 models AUC = 0.74. The InceptionV3 model demonstrates the best performance with an F1 score of 0.85. Recall scores generally ranged between 0.62 and 0.85. **Conclusions**: In our study, hyperdensity areas in initial NCCT images obtained after EVT in AIS patients were successfully differentiated from HT and CA with high accuracy using CNN architectures. Our findings may enable the early identification of patients who would benefit from anticoagulation or antiplatelet therapy to prevent re-occlusion or progression after EVT.

## 1. Introduction

Acute ischemic stroke (AIS) is the loss of blood flow to a specific part of the brain due to the occlusion of a cerebral artery [1,2]. It is associated with high morbidity and mortality worldwide [1,3]. The primary treatment for AIS involves reopening the blocked vessels to salvage the tissue in the ischemic penumbra [4]. Before the 1990s, AIS treatment relied on symptomatic management; however, the approval of intravenous tissue plasminogen activator (IV-tPA) by the Food and Drug Administration (FDA) in 1995 formed the basis of AIS treatment for nearly 20 years [5,6]. However, the late presentation of stroke patients to the hospital highlighted the need for new treatment methods [5,7]. Endovascular treatment (EVT) has become a major option for the treatment of large vessel occlusion (LVO) strokes in the last decade [7,8,9]. Randomized controlled trials have confirmed the benefits of using EVT in the treatment of stroke patients compared to those receiving only standard medical care [9,10,11]. Considering the demonstration of EVT efficacy in AIS patients and the widening and increasing focus of research in this field, the future of post-EVT management is undoubtedly advancing rapidly [12]. Early hyperdense areas are common on non-contrast computed tomography (NCCT) of the brain after EVT; however, their relationship with clinical outcomes and EVT treatment has not been clearly assessed [1,2,13]. It is assumed that these hyperdense areas are related to the increased permeability of the blood–brain barrier (BBB) in sensitive brain tissue that has undergone infarction, which can be described as the ‘contrast enhancement phenomenon’, and that allowed the contrast used during the EVT procedure to be visualized outside the normal cerebral vascular system [1,13,14]. The identification and characterization of hyperdense areas play a critical role in determining the clinical status and guiding the treatment of patients with acute ischemic stroke (AIS). Following EVT, hyperdensities observed on NCCT scans may result from either contrast accumulation (CA) or hemorrhagic transformation (HT) [14,15]. Differentiating these two entities is essential, as the clinical approach significantly varies depending on the underlying cause [16]. In some cases, the initiation of anticoagulation or antiplatelet therapy—potentially beneficial after EVT—may be postponed until intracerebral hemorrhage is definitively excluded to avoid the risk of reocclusion or hemorrhagic progression [15,16]. Therefore, the accurate identification of post-EVT hyperdensities is crucial for optimizing clinical outcomes [13,15].

This study aimed to correctly interpret hyperdense areas on early brain NCCT images of patients with AIS undergoing EVT procedures. Thus, it aims to provide a more effective and safe approach in treatment planning and patient follow-up in the stroke unit after the procedure by early hemorrhage detection. In this context, deep learning-based artificial intelligence (AI) models were used in this study for the differential diagnosis of hyperdense areas after EVT.

## 2. Materials and Methods

Ethics: This single-center retrospective study was conducted in the radiology clinic. Consent was obtained from all participants in this study. The study was approved by the Ethical Review Committee (Decision number: 2025/07).

### 2.1. Data Set Characteristics

#### 2.1.1. Patient Selection

Between January and December 2024, patients who presented to the clinic with a diagnosis of AIS and underwent mechanical thrombectomy (MT) due to LVO were evaluated. Endovascular treatment was applied to stroke patients with an Alberta stroke program early CT score (ASPECTS) > 6, a pre-procedural National Institutes of Health Stroke Scale (NIHSS) score > 6, and symptoms that started less than 6 h before treatment. All patients were selected for endovascular recanalization treatment according to the current guidelines from the American Heart Association/American Stroke Association for stroke patient management [17].

Patients aged 18 years or older who underwent acute EVT for LVO with successful recanalization were included in the study if they demonstrated hyperdensity in the corresponding perfusion territory on NCCT within 24 h post-procedure and had follow-up NCCT imaging available. Exclusion criteria were as follows: patients with posterior system occlusion, patients undergoing stenting of the extracranial internal carotid artery during EVT therapy or stenting in the case of intracranial atherosclerotic disease, patients without hyperdensity in the NCCT after EVT, patients without follow-up NCCT, patients not followed up in our clinic, patients lost to follow-up in the early period, and patients whose NCCT examinations were not of diagnostic quality.

Aside from these, the images assessed in the follow-up NCCT scan were excluded, as evaluated below.

1. New emerging densities were evaluated as hematoma developing after 24 h.
2. The presence of a hematoma was identified; however, cases with a hyperdense size that did not diminish according to the first screening of NCCT but decreased by more than 50% were evaluated as hematoma and CA together.
3. Sequential imaging, images where the decreasing component of the hyperdense area was greater than the increasing component, were evaluated as CA and HT together.

The exclusion and inclusion criteria were evaluated in 52 patients (21 female and 31 male) who were admitted to our Tertiary Stroke Center and met the criteria, were included in this study, and analyzed retrospectively.

Basin characteristics, demographic information, stroke features, and pharmacological treatment data were collected at admission. Patients with occlusion sites of the internal carotid artery (ICA), middle cerebral artery (MCA) M1, MCA M2, and ICA + MCA were included. EVT results were determined at the end of the procedure using digital subtraction angiography according to the modified thrombolysis in cerebral infarction (mTICI) revascularization scale. These results were assessed as complete to incomplete recanalization (mTICI 2a-3) or no recanalization (mTICI 0-1) [18].

#### 2.1.2. Image Acquisition

All patients underwent NCCT (initial scan) within the first 24 h post-EVT procedure and NCCT (follow-up scan) after the first 24 h. The initial scan and follow-up scan NCCT were performed using a 16-slice CT scanner (Toshiba Alexion Advance Edition 16, Tokyo, Japan) with a contiguous axial plane of 5 mm, parallel to the inferior orbitomeatal line, from the base of the skull to the apex. The scanning parameters were as follows: 120 kVp, 270 mAs, 1 × 5 mm collimation, 0.4 s/r rotation time.

#### 2.1.3. Imaging Evaluation

**Figure 1:**
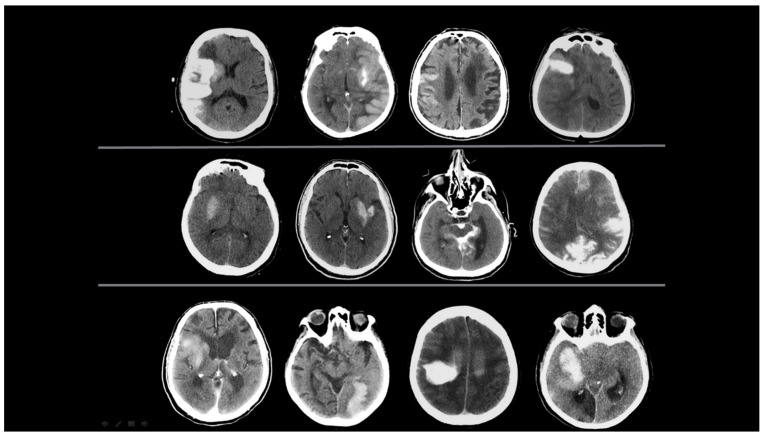
Initial NCCT scans from 12 different patients. Hyperdense areas observed within the first 24 h after EVT are presented, specifically located in the regions where EVT was performed. Two radiologists evaluated changes in the location, size, morphology, and density of these hyperdense areas. Each characteristic was categorized as increasing, decreasing, unchanged, or resolved.

Patients with hyperdensity areas found on NCCT tests within the first 24 h after EVT were designated as having ‘initial NCCT scan’ examinations. NCCT images obtained 24 h after the EVT procedure were designated as ‘follow-up NCCT scans’. These images were analysed retrospectively. The initial and follow-up NCCT images after the EVT procedure were evaluated through a consensus review by two radiologists from the team that performed the EVT procedure (the team leader is an interventional neuroradiologist with 10 years of experience (M.B.) and a board-certified radiologist (E.K.). An example of the evaluated initial NCCT scan images is presented in Figure 1.

At the end of the evaluation, radiologists labeled the hyperdensity areas in the initial NCCT in two different ways according to the following assessment.

1. Contrast Accumulation: The first scan found in the NCCT and the follow-up scan in the NCCT met all of the following conditions;
  1. Sequential follow-up scan showed hyperdensity that completely disappeared without leaving a hematoma area.
  2. Sequential follow-up scans show no newly developed hyperdense area.
  3. In sequential follow-up scans, hyperdensity that gradually decreases and completely disappears in NCCT images after 72 h.
2. Hemorrhagic Transformation: The initial scan shows NCCT, and the follow-up scan shows NCCT meeting any of the following conditions.
  1. Hyperdensity that does not decrease or remains constant.
  2. Increase in hyperdensity size.

**Figure 2:**
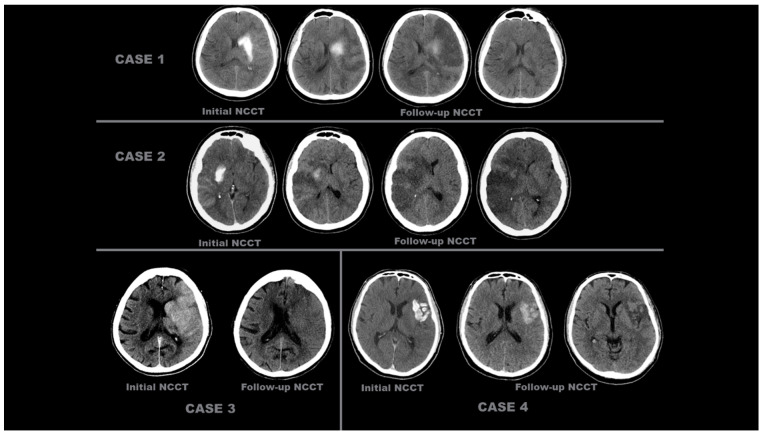
Initial and follow-up NCCT scans of four different patients with ‘contrast accumulation’. CASE 1: A 55-year-old male patient was evaluated with initial NCCT within the first 24 h after complete recanalization (TICI 3) following occlusion of the left MCA M2. Hyperdense areas seen at the level of the left caudate nucleus and lentiform nucleus decreased and disappeared on subsequent follow-up NCCT scans taken 24 h later. CASE 2: A 38-year-old female patient, after complete recanalization (TICI 3) following right ICA occlusion, during the initial NCCT examination within the first 24 h, showed more pronounced hyperdensity areas at the level of the right lentiform nucleus and in the MCA perfusion area cortex, which decreased and disappeared in the follow-up NCCT scans 24 h later. CASE 3: An 85-year-old female patient underwent EVT, which was completed with complete recanalization (TICI 3) after left ICA occlusion. The initial NCCT scan performed within the first 24 h showed hyperdense areas in the cortical territory of the left MCA, which disappeared in the follow-up NCCT scan taken 24 h later. CASE 4: A 52-year-old male patient presented with complete recanalization (TICI 3) after EVT due to occlusion of the superior trunk of the left MCA M2 segment. In the NCCT scan (initial NCCT) performed within the first 24 h after the procedure, an area of hyperdensity in the left insular cortex and its lateral region decreased and resolved in the subsequent follow-up NCCT scans taken 24 h later.

**Figure 3:**
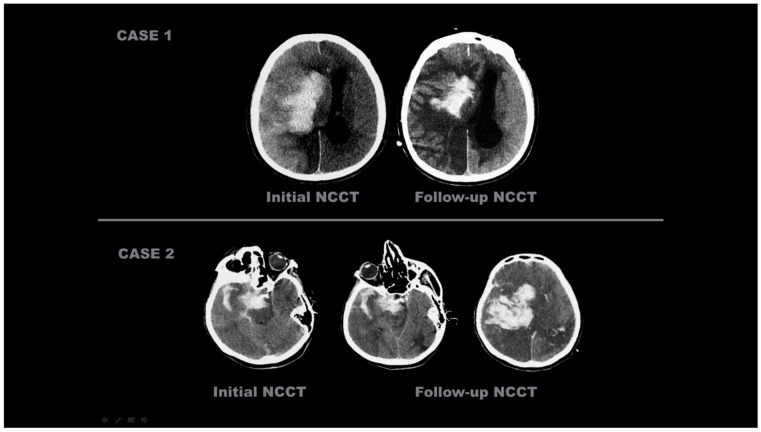
Initial and follow-up NCCT scans of four different patients with ‘hemorrhagic transformation’. CASE 1: An 86-year-old male patient, after EVT was completed with complete recanalization (TICI 3) following right ICA occlusion, underwent an initial NCCT examination within the first 24 h, which showed hyperdense areas in the right basal ganglia, insular cortex, and MCA watershed area in the cortex. These hyperdense areas were still present on the 38-h follow-up NCCT scan. CASE 2: A 78-year-old female patient with right MCA M2 occlusion; the initial NCCT examination taken within the first 24 h after EVT concluded showed areas of hyperdensity in the right temporal lobe, in the subarachnoid space, and in the tentorium, which were still present 32 h after the NCCT scan.

The representative images of the labeled patients evaluated by radiologists are shown in Figure 2 and Figure 3.

### 2.2. Dataset Features

**Figure 4:**
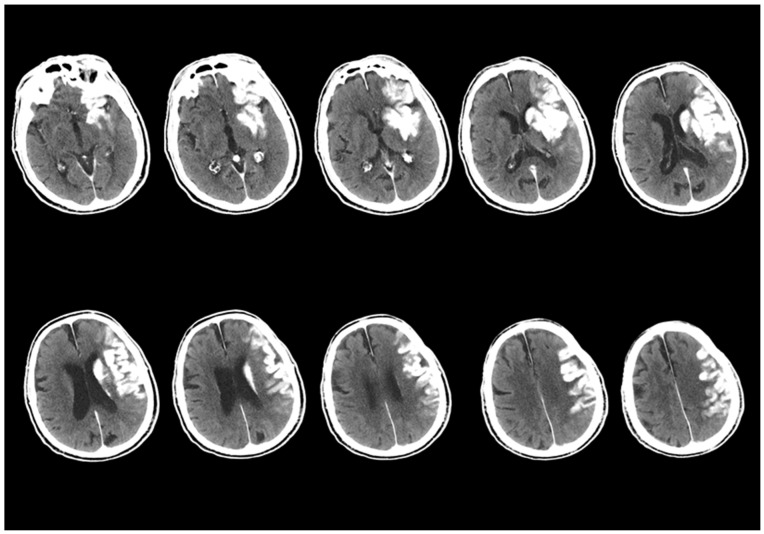
An example of the first scan used in the dataset, the NCCT scan, is shown. The images from the NCCT examination taken within the first 24 h after the EVT procedure of an 87-year-old male patient with a left MCA M1 occlusion include images obtained from approximately 5–6 slices above and below the slice where the hyperdense area is best visualized, with a 5 mm slice acquisition interval.

In the study, the first screening of NCCT examinations after EVT procedures was evaluated. The initial NCCT tests were classified into two different groups as ‘contrast accumulation’ or ‘hemorrhagic transformation’ as mentioned above. A dataset containing images in JPEG format and with a resolution of 1920 × 1080 pixels was used for the study. This dataset includes approximately 556 images obtained from a total of 52 different patients. There is an equal number of image data and patient counts in both groups. To enhance classification performance and prevent unnecessary repetitions in the dataset, only slices where the hyperdense area was most clearly visible were selected. During this selection process, slices were included in addition to the slice where the hyperdense area was most intensely located for each patient, taking approximately 5–6 slices above and below. The remaining excess data were removed, making the dataset more focused. The dataset for all patients consisted of approximately 9–13 slices where the hyperdense area was most clearly visible, with a slice acquisition interval of 5 mm. An example of the files composed of these images is shown in Figure 4. Subsequently, the images of all patients were organized and filed separately for each patient.

#### 2.2.1. Image Preprocessing

The raw data used in the study consists of high-resolution images with a resolution of 1920 × 1080 pixels. However, in order to increase data consistency during the analysis process, optimise processing time, and ensure that the models operate more efficiently, these images were standardised to a size of 640 × 640 pixels using specific focus slices. This resizing process was performed by focusing on the pathological areas of the images, and only the most meaningful sections of each image were selected for analysis.

The images were standardised to a dimension of 640 × 640 pixels prior to conversion into a suitable format for deep learning models. In this process, a 32-core convolution operation was applied. The result of this operation was the acquisition of feature maps of size 640 × 640 × 32, corresponding to each input image.

#### 2.2.2. Labeling and Data Augmentation

After the preprocessing steps were completed, the images were classified, and the corresponding class file names were divided into train, validation, and test. In this classification process, the dataset distribution was made in such a way that the highest percentage in each patient class would be trained, specifically at rates of 70%, 20%, and 10%. After the separation process, the train folder was allocated to correspond to 70% of the total number of patients. During the training phase of the AI, the dataset was balanced with 186 images in the CA class and 186 images in the HT class.

In the validation folder, a dataset was created corresponding to 20% of the number of patients, and in the test folder, a dataset was created corresponding to 10% of the total number of patients, with separate datasets for the CA class and HT classes. Due to the relatively small sample size in the image processing and AI

Data augmentation operations were randomly applied using the PIL (Python Imaging Library—Pillow 10.3.0)) in the Python programming language. The PIL library offers various functions commonly used in the image data generation process for machine learning, such as rotation, flipping, zooming, scaling, cropping, brightness enhancement, and shifting. In this study, standard image augmentation methods were applied to enhance the model’s generalization performance and to ensure the diversity of the training data. The applied operations included blurring, cropping (zooming 0–20%), rotation (between −15° and 15°), and adding noise. Additionally, to strengthen the generalization capability, these morphological operations were applied together in some cases. For example, both flipping and adding noise were applied to an image simultaneously.

**Figure 5:**
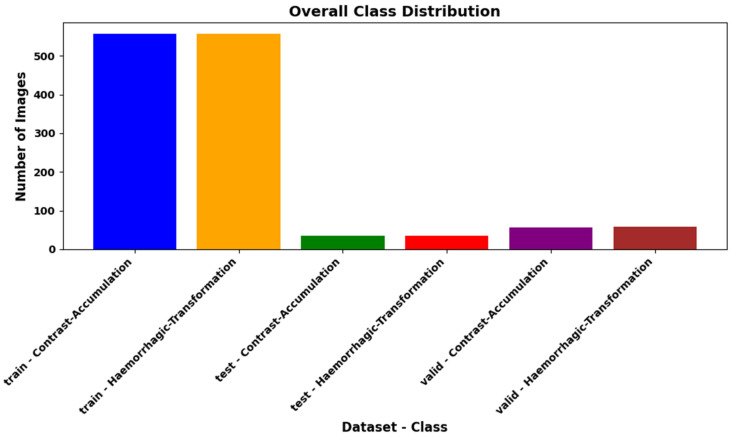
In the AI training process of the study, the expanded dataset obtained after applying the 3-fold data augmentation operations to the training portion has been divided into three sub-sections after completing the process without augmentation in other datasets. These sections have been created to ensure a balanced distribution of the number of data points for each class.

As a result of these augmentation operations, the training data set was increased threefold, rising from 186 to 558. In the CA class, 558 images were generated, and in the HT class, 558 images were created. A total of 1116 images were used for training. The validation process was completed with 115 images, and the testing process was completed with 69 images. In the final version of the data set, 1300 images were used. This process significantly contributed to the diversification of the data set and increased the model’s ability to adapt to different variations during training. Figure 5 presents the number of data points in each sub-section obtained after this operation and analyzes the data content.

#### 2.2.3. Transfer Learning and Used Models

Transfer learning is the process of applying the knowledge acquired from a model previously trained on large datasets (such as ImageNet) to a new problem. In this study, DenseNet201, InceptionResNet, InceptionV3, NASNetLarge, ResNet50, ResNet101, VGG16, VGG19, and Xception architectures have been tested as the base model. These architectures effectively learn features at various scales by using convolutional kernels of different sizes within the same layer. Furthermore, the code infrastructure has been designed to easily integrate with different models. For example, alternative models, such as ResNet50 and VGG16, can be integrated into the same structure instead of InceptionV3. Each model provides a strong foundation for low-level feature extraction due to the pretrained weights (weights = ‘magenet’). However, the upper layers have been restructured to provide a fit specific to the classification task.

#### 2.2.4. Model Architecture

**Figure 6:**
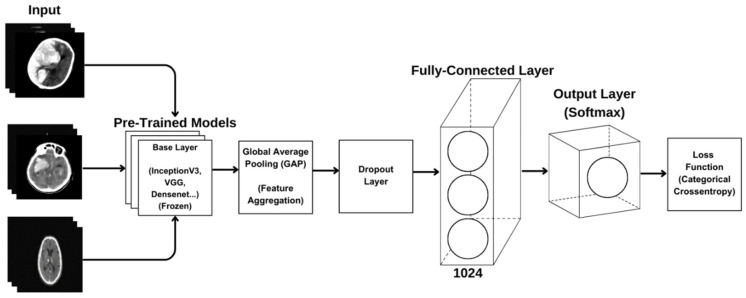
Created CNN model architecture.

The transfer learning-based model architecture aims to leverage the knowledge of a pre-trained model to achieve better performance on new tasks. This architecture begins with a base layer that facilitates low-level feature extraction and includes a Global Average Pooling (GAP) layer that summarizes convolutional feature maps to reduce the risk of overfitting. Key components also include fully connected layers that enhance classification performance, linear combinations that provide a higher-level representation of learned features, and the Rectified Linear Unit (ReLU) that aids in learning nonlinear relationships. Additionally, a Dropout Application is employed to prevent overfitting, and the Softmax activation function is used in the output layer to calculate probability distributions among classes. Together, these elements interactively contribute to improving the overall success of the model and enable more accurate predictions. This structure effectively utilizes the advantages of transfer learning to present an efficient and effective classification process. All model architectures are included in the diagram shown in Figure 6.

#### 2.2.5. Model Training

In our study, images related to ‘Hemorrhagic Transformation’ and ‘Contrast Accumulation’ diseases were used for training the model. The images were classified according to the file name for each class and organized into three main categories: ‘train’, ‘validation’, and ‘test’. 70% of the dataset was allocated for training, 20% for validation, and 10% for testing. To accelerate the training process, an NVIDIA RTX 4090 GPU was used, and CUDA functionality was enabled. The CUDA version used in the study was 1.12.x, and all operations were performed on the GPU, significantly reducing the training time. TensorFlow 2.6.0 and Keras libraries were used for training the model. The training parameters are provided in Table 1. Hyperparameter settings are set under the same training procedures to ensure a fair comparison across all models.

The size of the visuals determined during the training of the models is 640 × 640 pixels. Having visuals at this size ensures the preservation of details while keeping the computational load at a reasonable level. During the training process, 32 visuals were sent to the model in each iteration, thus optimizing the training time by effectively using GPU memory. The base of the model includes different pre-trained models, such as DenseNet201, InceptionResNet, InceptionV3, NASNetLarge, ResNet50, ResNet101, VGG16, VGG19, and Xception. These base models are trained on large datasets, such as ImageNet, and provide high accuracy in visual feature extraction. By testing different models, the base model that provides the best performance will be determined based on the analysis results.

In the feature extraction layer, GAP has been applied, thus reducing the size of feature maps, decreasing the number of parameters, and minimizing the risk of overfitting. After the GAP layer, a Dense layer with 512 neurons has been used, employing ReLU activation and Dropout (0.5) to enhance the model’s generalization performance. In the classification layer, the Softmax activation function has been used for multi-class predictions. The Adam algorithm has been preferred for model optimization, and Categorical Crossentropy has been used as the loss function. The training process has been carried out by presenting the dataset to the model for 40 epochs. This structure allows the model to learn quickly and effectively while preventing overfitting and increasing generalization success.

#### 2.2.6. CNN Model Analyses

The performance of CNN models after the training process is evaluated using various metrics for the object classification task. Accuracy expresses the ratio of the total number of examples correctly classified by the model. The loss function measures the difference between the values predicted by the model and the actual values. As the loss value decreases, the performance of the model increases. Categorical cross-entropy is commonly used in CNN models. Validation accuracy is the rate of correct predictions on the model’s validation dataset. It is used to test the model’s ability to generalize. Validation accuracy is checked against training accuracy to monitor for overfitting. Validation loss is the loss function calculated on the validation dataset. As a valid method among the validation metrics of AI analyses, the F1 score was calculated on the test set to evaluate the model’s capacity for making correct predictions and how well it identified positive examples. The F1 score is defined as the harmonic mean of the precision and recall metrics, and it is particularly used in cases with imbalanced datasets or where misclassifications are costly.

Precision is the ratio of truly positive predictions to the total number of positive predictions, and it is calculated as specified in Equation (1):

(1) $$Precision=\frac{TP}{TP+FP}$$

Recall is the ratio of correctly predicted positive samples, and it is calculated as specified in Equation (2):

(2) $$Recall=\frac{TP}{TP+FN}$$

In the equations, TP refers to true positive predictions, FP refers to false positive predictions, and FN refers to false negative predictions. Especially in classification problems, a high F1 score indicates that the model has both a high capacity for making correct predictions and a low rate of missing positive examples.

Additionally, Receiver Operating Characteristic (ROC) curves were used to visually evaluate classification performance by examining the relationship between TPR (True Positive Rate) and FPR (False Positive Rate) according to different threshold values of the trained models. The ROC curve measures how well the trained models can distinguish between positive and negative classes. AUC (Area Under the Curve) represents the area under the ROC curve and is used to assess a model’s classification performance. AUC = 1.0: Perfect classifier (correctly separates all positive and negative examples). AUC = 0.5: A model that predicts randomly (no separation ability). AUC < 0.5: A poor model (classifies negatives as positives and positives as negatives). The equations provided in Equations (3)–(5) are used when plotting the ROC curve and calculating the AUC value.

(3) $$TPR=\frac{True\;Positives(TP)}{Total\;Positives(P)}$$

(4) $$FPR=\frac{False\;Positives(FP)}{Total\;Negatives(N)}$$

(5) $$AUC=\int_{0}^{1}TPRd\left(FPR\right)$$

The performance of the confusion matrix analyzed DenseNet201 and Inceptionv3 models were compared in detail using confidence interval analysis and McNemar test (*p*-value analysis).

Confidence interval is a statistical measure that indicates the interval within which the measured performance value of a model lies with a certain probability (usually 95%). In this analysis, 95% confidence intervals were calculated for the accuracy rates of the InceptionV3 and DenseNet201 models using the Wilson score interval method. The Wilson confidence interval gives more reliable results compared to classical Binomial Confidence Interval methods, especially in small data sets or when outliers are effective.

The McNemar test is a statistical test that assesses whether there is a significant difference between matched data sets. This test is particularly suitable for determining whether there is a significant difference in the accuracy of two models tested on the same data set.

## 3. Results

In the study, data from 52 patients (21 female and 31 male) were obtained, consisting of 26 patients from the CA group and 26 patients from the HT group, resulting in a dataset of 556 images. A total of 186 images for CA and 186 images for HT were used for training (increased to 558 images), while 115 images were used for validation data and 69 images were used for testing data.

The results of metrics evaluated after training the models created with the CNN structure, including accuracy, loss, validation accuracy, and validation loss, are presented in Table 2 and Table 3.

Looking at Table 2, the NASNetLarge model showed the highest performance on the training set, with an accuracy of 91.7%, an F1 score of 90.2%, precision of 93%, and recall of 92%. Similarly, the DenseNet201 model achieved an accuracy of 88.3% and an F1 score of 79, while the Xception model displayed an accuracy of 87.7% and an F1 score of 87. On the other hand, ResNet101 and ResNet50 stand out, with ResNet101 showing an accuracy of 51.9% and an F1 score of 57, and ResNet50 achieving an accuracy of 53.3% and an F1 score of 52. The VGG16 and VGG19 models also demonstrated performance with accuracy rates of 63.3% and 58.3%, respectively.

**Figure 7:**
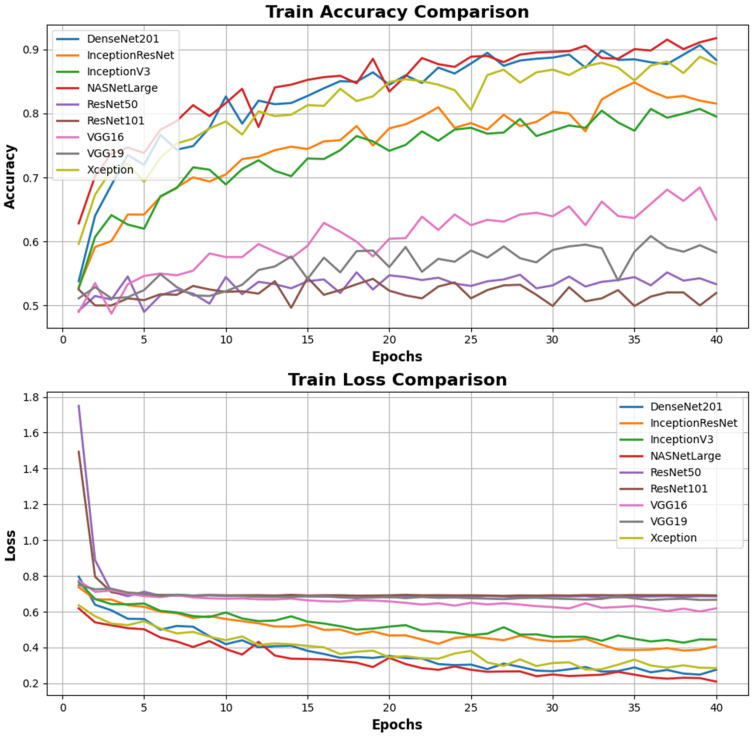
Comparison of accuracy and loss performance in the training processes of different deep learning models. The Train Accuracy Comparison graph shows that the DenseNet201 model exceeds the validation value range and achieves the highest accuracy rate, surpassing other models, despite the NASNetLarge values due to breaching acceptable ranges in validation losses. The InceptionV3 model also stands out with high accuracy values. However, the ResNet101, VGG16, and VGG19 models perform weakly compared to other models with low accuracy rates. The Train Loss Comparison graph indicates the training losses. The DenseNet201 and NASNetLarge models demonstrate the most efficient learning process by achieving the lowest loss values. Conversely, the ResNet101 and VGG19 models exhibit weak training performance with high loss values. Notably, although the training loss of the VGG19 model shows a decline over epochs, it remains behind the other models.

**Figure 8:**
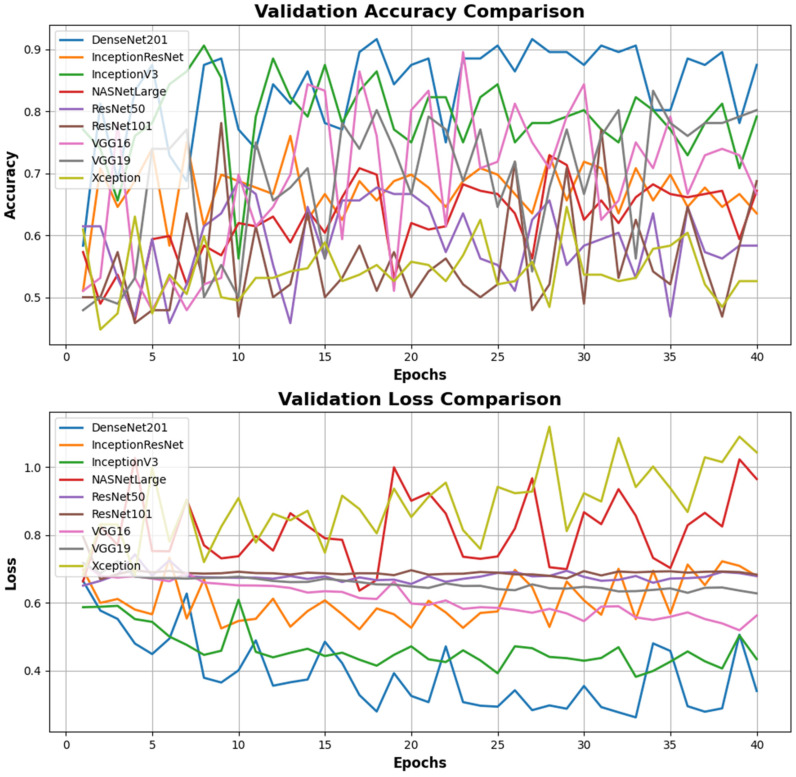
Comparison of accuracy and validation loss performance according to the validation data in the training processes of different deep learning models. In the Validation Accuracy Comparison graph, the DenseNet201 model achieves the highest validation accuracy overall, demonstrating a more consistent performance than other models. The NASNetLarge and InceptionV3 models also show strong performance in terms of validation accuracy. In contrast, the validation accuracy of the ResNet50, ResNet101, and VGG19 models is quite fluctuating and remains at low levels. In the Validation Loss Comparison graph, the DenseNet201 model achieves the lowest validation loss, indicating effective learning during the validation process. The NASNetLarge model also exhibits good performance with low loss values. Conversely, the validation loss of the ResNet50, ResNet101, and VGG19 models remains high and follows an inconsistent trend throughout the epochs.

**Figure 9:**
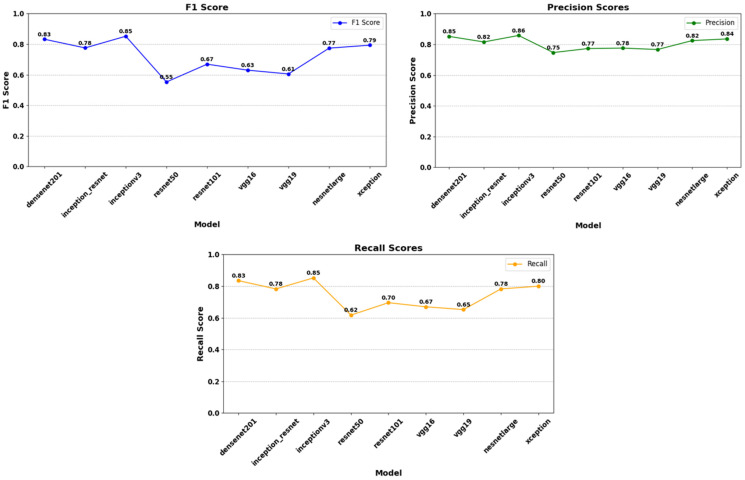
Analysis of F1 score, precision score, and recall score according to the model’s test dataset. The InceptionV3 model demonstrates the highest performance with an F1 score of 0.85. It is followed by DenseNet201 (0.83), while the Inception_ResNet and NasNetLarge models exhibit similar performances with F1 scores of 0.78 and 0.77, respectively. However, the ResNet50 model shows the lowest performance with an F1 score of 0.55. The Xception model achieves an F1 score of 0.79, indicating medium-level performance. In this context, choosing models that achieve high F1 scores, such as InceptionV3 and DenseNet201, provides more reliable results in classification problems. The highest Precision score is obtained by the InceptionV3 model (0.86), indicating that this model has the highest true positive rate. In contrast, the lowest Precision score belongs to the ResNet50 model (0.75), suggesting that this model produces more false positives compared to others. Precision scores generally range from 0.75 to 0.86, with most models exhibiting a performance of 0.77 or above. The highest recall score is achieved by the InceptionV3 model (0.85), indicating that this model has the highest rate of correctly identifying positive instances. Conversely, the lowest recall score belongs to the ResNet50 model (0.62), suggesting that this model misses many positive instances. Recall scores range from 0.62 to 0.85, with models such as InceptionV3 and DenseNet201 showing high performance, while ResNet50 and VGG16 models perform lower. Notably, DenseNet201 (0.83) and Xception (0.80) stand out with balanced and high sensitivity rates.

**Figure 10:**
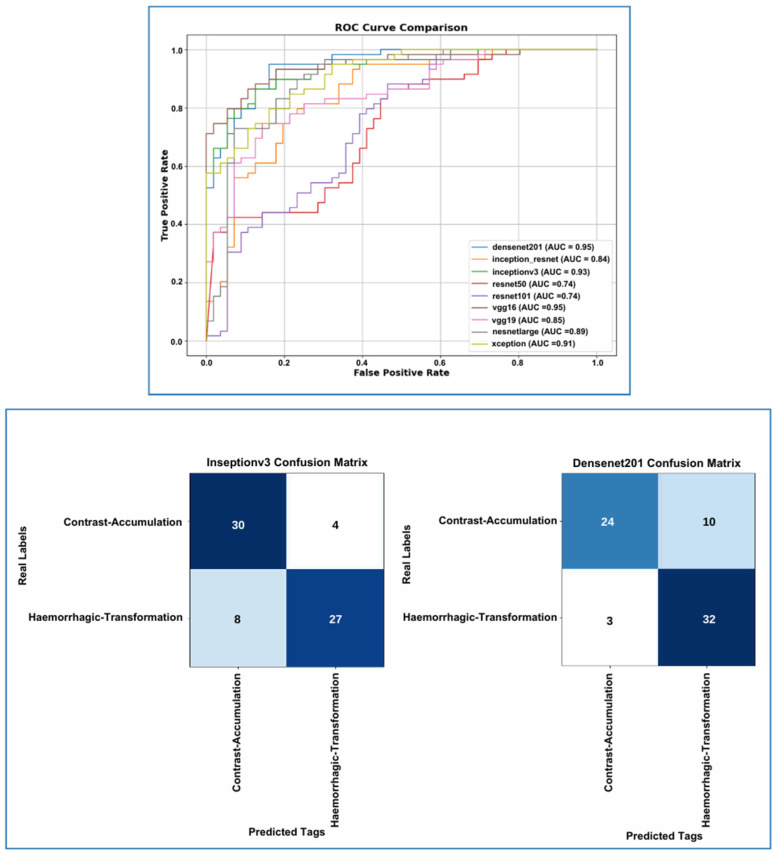
On the test set, DenseNet201 achieved the highest AUC (0.95), followed by InceptionV3 (0.93), Xception (0.91), and NasNetLarge (0.89). DenseNet201 showed superior sensitivity in detecting hemorrhagic transformation with a low false negative rate, though it had lower precision in the Contrast Accumulation class due to more false positives. InceptionV3, on the other hand, provided better precision in the contrast accumulation class but showed reduced sensitivity for hemorrhagic transformation. Model selection should depend on clinical priorities, favoring InceptionV3 for precision in CA and DenseNet201 for sensitivity in HT.

According to the results in Table 3 from the test set, DenseNet201 emerges as the highest performing model, with an accuracy of 85.3%, a loss of 0.253, an F1 score of 0.83, precision of 0.85, and sensitivity of 0.83. The InceptionV3 model performed second best, with an accuracy of 83.2% and a loss of 0.321. This model had an F1 score of 0.85 and a precision value of 0.86, particularly achieving better results than DenseNet201 in terms of the precision metric. A comparison of the models’ accuracy and loss performance was made, with the analysis of the training dataset shown in Figure 7 and the analysis of the validation dataset shown in Figure 8. The values analyzed for the precision score, as specified in Equation (1), and the recall score values analyzed as specified in Equation (2) are presented in Figure 9. The analysis provided in Figure 10 assesses the test dataset, with a total of 34 images in the CA class and a total of 35 images in the HT class. The top-performing models, DenseNet201 and InceptionV3, were evaluated, and their success rates were analyzed using confusion matrices.

The performance of DenseNet201 and Inceptionv3 models, confidence interval analysis, and McNemar’s test (*p*-value analysis) are presented in Table 4.

## 4. Discussion

Intra-arterial recanalization is the fundamental step in the treatment of AIS patients [16]. EVT has rapidly developed over the last 10 years and is an effective treatment for AIS associated with LVO occlusion [5,7]. MT is a procedure in which catheters are directed to the location of the blood clot in the brain through the patient’s arteries. Various contrast agents are used to enhance the visibility of vessels during the EVT procedure [7]. The neurotoxicity caused by the contrast agent can damage the BBB and may lead to extravasation [19]. If only endothelial damage occurs, it may indicate the presence of contrast without hemorrhage within hyperdense areas [13,19,20,21]. New hyperdense areas are frequently seen on NCCT images following EVT. Hyperdense areas do not often indicate hemorrhage [13]. The contrast agent affects the brain parenchyma for at least 24 h and diminishes over time [19,20]. However, hemorrhage may appear after 24 h and on follow-up imaging [19,20,21,22,23]. In many studies, hyperdense areas observed on NCCT examinations following MT have been described using terms such as “contrast enhancement”, “contrast staining”, or “contrast extravasation”, leading to a lack of standardization due to different nomenclatures [13]. ‘Contrast extravasation’ may later refer to mixed hyperdensity, which is a clear indicator of a certain degree of bleeding in addition to contrast [24]. Due to the interchangeable use of the terms and the confusion of meaning it creates, we used the term ‘contrast accumulation’ in our study. We preferred this term for hyperdensity areas seen in NCCT examinations within the first 24 h post-MT, where all hyperdensity areas disappeared in control NCCT examinations and in images without hemorrhage. Regardless of terminology, the goal is to predict the progression of NCCT hyperdensity post-MT and the possibility of it leading to a condition evaluated as hemorrhage. This is critical because early differentiation between CA and HT in AIS patients who have undergone MT is crucial for applying antiplatelet agents at the earliest stage [24,25,26]. Identifying patients who will benefit from anticoagulation or antiplatelet therapy post-procedure is necessary to prevent re-occlusion/progression, and delayed treatment in the timeframe needed to rule out HT [26]. Therefore, many studies have been conducted to better characterize this radiographic finding regarding its potential impact on prognosis [1,23,27,28,29]. However, in conventional non-contrast CT, it is difficult to distinguish hemorrhage from contrast extravasation in the early stages after EVT due to the similarities in Hounsfield densities of iodine and hemorrhages [25,26]. Additionally, studies have assessed the differentiation of hyperdensity at the earliest stage using advanced magnetic resonance imaging techniques or dual-energy computed tomography (DECT) and its relationship with prognosis at a later stage [1,2,19,30,31]. However, these studies have shown that there is currently no adequate imaging technique developed to make this critical differential diagnosis, which is essential for the clinical recovery of AIS patients, with high accuracy, and that many new and broadly scoped studies are needed in this area [31]. Moreover, the deployment of advanced MR techniques and DECT is both costly and challenging due to the complexity of the equipment and the necessity for specialist expertise. However, in view of the increasing prevalence of AIS, there will be a greater demand for more practical and accessible techniques. However, the evaluation of radiological imaging findings with AI has gained importance in recent years [32]. Many studies have shown that AI makes diagnosis quick and easy and may improve the prognosis of many diseases [33,34,35].

This study showed that deep learning models could accurately predict CA and HT from NCCT of the brain after intra-arterial recanalization treatment in AIS patients. In the context of the training and validation processes of our study, the DenseNet201 model has demonstrated optimal performance, as evidenced by its superior values within the acceptance ranges of the metrics. This model distinguishes itself through its notable accuracy of 0.883, its minimal loss of 0.275, and its maximum F1 score of 0.83. The balanced values of precision (0.85) and recall (0.83) further demonstrate the model’s efficacy in identifying true positives and making accurate positive predictions.

InceptionV3 and NASNetLarge models demonstrate impressive performance. In particular, InceptionV3 demonstrates notable efficacy, attaining an F1 score of 0.85 and exhibiting balanced precision-recall values. However, the validation metric value of the NASNetLarge model exceeds the validation range, and the high validation loss (0.965) indicates that the model’s generalization capacity can be questioned for optimal performance. However, VGG16 has shown a more balanced performance compared to VGG19. Finally, the Xception model presents average performance. Although the model’s F1 score (0.79) and recall (0.80) are at acceptable levels, the high validation loss (1.04) affects the model’s overall capacity. In conclusion, it is observed that models with deep and complex architectures (e.g., NASNetLarge and DenseNet201) generally provide superior results, while older and simpler architectures demonstrate limited performance with modern datasets. DenseNet201 and InceptionV3 were not only compared based on a basic metric, such as accuracy. More comprehensive metrics, such as F1 score, precision, recall, and AUC scores, were also considered. For these metrics used in decision making, the F1 score takes precedence. In unbalanced datasets (e.g., rare diseases), the F1 score is often the most important metric. This is because it balances both precision and recall, i.e., it increases the number of true positives while trying to reduce the number of false negatives. This is critical in applications where both errors need to be minimized. In addition, the hyperparameters used to train the models are consistent and fair. Both models have been trained under the same conditions.

In the literature, You et al. differentiated CA from HT using MRI, specifically an immediate post-interventional diffusion-weighted imaging (DWI) protocol that included gradient-recalled-echo (GRE) sequences [30]. Additionally, Tijssen et al. investigated the utility of dual-energy CT (DECT) in distinguishing between CA and HT, evaluating 22 patients in their study. They reported a positive predictive value (PPV) of 100% for detecting hemorrhage with DECT, along with a negative predictive value (NPV) of 89% and an overall diagnostic accuracy of 89% [36]. To the best of our knowledge, our study is the first to utilize deep learning for distinguishing CA from HT in AIS patients based on initial NCCT images. According to our findings, certain CNN architectures show considerable promise in this field.

While this study demonstrates promising results in differentiating hemorrhagic transformation from contrast accumulation using deep learning, several limitations should be acknowledged. First, the retrospective design and single-center dataset with limited sample size may affect generalizability. Second, while expert-annotated ground truth was prioritized, model interpretability techniques, such as Grad-CAM, were not employed. Third, the evaluation was restricted to included cases, and external validation was not performed. Additionally, although class imbalance was addressed, the natural prevalence in our data may impact clinical applicability. Finally, while established CNN architectures were selected based on medical imaging performance, newer models, such as transformers, were not explored. These factors highlight the need for future multicenter studies with larger datasets, external validation, and enhanced model evaluation.

## 5. Conclusions

The present study constitutes a piece of original research, the aim of which was to classify hyperdense areas in NCCT images performed after EVT in AIS patients. This classification is informed by a limited body of literature that has been analyzed with deep learning-based models. The approach proposed in this study is capable of differentiating between HT and CA in NCCT after MT with a high degree of accuracy.

The findings of the present study offer a promising approach for the management of patients who require anticoagulation or antiplatelet therapy after EVT. The study also provides valuable data that could drive innovative research in this area, requiring further studies in the future.

## Figures and Tables

**Table 1:** Training parameters of the CNN model.

Image Size	Batch Size	Base Model	GAP	Dense	Output Layer	Optimizer	Loss Function	Epoch
(640,640)	32	DenseNet201 InceptionResNet InceptionV3 NASNetLarge ResNet50 ResNet101 VGG16 VGG19 Xception	GlobalAvere Pooling2D	512 Neurons ReLU activation Dropout(0.5)	Softmax	Adam	Categorical Crossentropy	40

**Table 2:** Performance analysis of the training set in training CNN models.

Models	Accuracy (0.7–0.9+)	Loss (0–0.5)	Valiation Accuracy (0.7–0.9)	Validation Loss (0–0.5)	F1 Score (0.7–1)	Precision (0.7–1)	Recall (0.7–1)
DenseNet201	0.883	0.275	0.875	0.339	0.79	0.80	0.79
InceptionResNet	0.815	0.406	0.635	0.6	0.81	0.83	0.81
InceptionV3	0.795	0.444	0.791	0.43	0.80	0.80	0.80
NASNetLarge	0.917	0.209	0.671	0.965	0.92	0.93	0.92
ResNet50	0.533	0.686	0.583	0.67	0.52	0.65	0.58
ResNet101	0.519	0.691	0.687	0.682	0.57	0.57	0.57
VGG16	0.633	0.619	0.666	0.562	0.69	0.77	0.71
VGG19	0.583	0.666	0.802	0.62	0.54	0.64	0.58
Xception	0.877	0.285	0.526	0.93	0.87	0.87	0.87

**Table 3:** Performance analysis of the test set as a result of training CNN models.

Models	Accuracy (0.7–0.9)	Loss (0–0.5)	F1 Score (0.7–1)	Precision (0.7–1)	Recall (0.7–1)
DenseNet201	0.853	0.253	0.83	0.85	0.83
InceptionResNet	0.792	0.366	0.78	0.82	0.78
InceptionV3	0.832	0.321	0.85	0.86	0.85
NASNetLarge	0.823	0.214	0.77	0.82	0.78
ResNet50	0.543	0.645	0.55	0.75	0.62
ResNet101	0.554	0.706	0.67	0.77	0.70
VGG16	0.612	0.617	0.63	0.78	0.67
VGG19	0.659	0.683	0.61	0.77	0.65
Xception	0.827	0.219	0.79	0.84	0.80

**Table 4:** Confidence interval and *p*-value analysis results; According to the Wilson confidence interval calculations, the accuracy confidence intervals of the InceptionV3 and DenseNet201 models largely overlap. The *p* = 0.388 value obtained for the McNemar test is above the generally accepted significance level of 0.05, indicating that there is no statistically significant difference between the accuracy performance of the two models. These results show that DenseNet201 and InceptionV3 have similar accuracy performance.

Model	Confidence İnterval (95%)	*p*-Value
InceptionV3	(0.720, 0.898)	0.388
DenseNet201	(0.704, 0.886)	0.388

## Data Availability

The original contributions presented in this study are included in the article. Further inquiries can be directed to the corresponding author.

## Abbreviations

NCCT	Non-Contrast Computed Tomography (NCCT)
AIS	Acute Ischemic Stroke (AIS)
EVT	Endovascular Thrombectomy (EVT)
CNN	Convolutional Neural Network (CNN)
IV-tPA	Intravenous Tissue Plasminogen Activator (IV-tPA)
FDA	Food and Drug Administration (FDA)
LVO	Large Vessel Occlusion (LVO)
BBB	Blood–Brain Barrier (BBB)
AI	Artificial Intelligence (AI)
MT	Mechanical Thrombectomy (MT)
ASPECT	Alberta stroke program early CT score (ASPECTS)
NIHSS	National Institutes of Health Stroke Scale (NIHSS)
ICA	Internal Carotid Artery (ICA)
MCA	Middle Cerebral Artery (MCA)
mTICI	modified thrombolysis in cerebral infarction (mTICI)
PIL	Python Imaging Library (PIL)
GAP	Global Average Pooling (GAP)
ReLU	Rectified Linear Unit (ReLU)
ROC	Receiver Operating Characteristic (ROC)
TPR	True Positive Rate (TPR)
TP	True Positive (TP)
FPR	False Positive Rate (FPR)
FP	False Positive (FP)
AUC	Area Under the Curve (AUC)
CA	Contrast Accumulation (CA)
HT	Hemorrhagic Transformation (HT)
